# A survey of prolapse practice in UK women’s health physiotherapists: what has changed in the last decade?

**DOI:** 10.1007/s00192-015-2864-9

**Published:** 2015-10-17

**Authors:** Suzanne Hagen, Diane Stark, Isla Dougall

**Affiliations:** Midwifery and Allied Health Professions Research Unit, Glasgow Caledonian University, Glasgow, UK; Functional Bowel Service, Glenfield Hospital, University Hospitals Leicester, Leicester, UK; Department of Psychology, University of Glasgow, Glasgow, UK

**Keywords:** Evidence-based practice, Pelvic floor, Physiotherapy, Prolapse, Survey

## Abstract

**Introduction and hypothesis:**

Prolapse is a common female problem, and conservative treatments such as pelvic floor muscle training (PFMT) are important options for women. Evidence supporting the effectiveness of PFMT for prolapse has grown over the last decade, and it was hypothesised that practice and practice guidelines would have developed in line with the evidence. To assess this, up-to-date information about the practice of physiotherapists working in women’s health regarding their treatment of prolapse was required.

**Methods:**

An online survey sent to members of the Association of Chartered Physiotherapists in Women’s Health and the Chartered Physiotherapists Promoting Continence. Results were compared with those of an earlier survey undertaken in 2002.

**Results:**

A 49 % response rate was achieved. The majority of respondents were senior physiotherapists (55 %) and had worked in women’s health for more than 10 years. Respondents were treating significantly more women with prolapse than a decade before: 36 % vs 14 % treated more than 50 women per year in 2002 and 2013 respectively (*p* < 0.001). Individualised PFMT (93 %), lifestyle advice (92 %) and biofeedback-assisted PFMT (83 %) were the most common treatment elements, with four being the average number of appointments. Forty-eight percent had changed their practice as a result of recent research; however, scepticism amongst medics, the referral of women directly for surgery, and constraints on resources were thought to be barriers to wider implementation of the evidence of PFMT for prolapse.

**Conclusions:**

There has been uptake of evidence-based prolapse practice by UK specialist physiotherapists in the last decade. Further research targeting the implementation of this evidence would be valuable in addressing potential barriers, and in supporting the need for physiotherapy in the treatment of prolapse.

## Introduction

Pelvic organ prolapse is a common condition thought to affect 40 % of women over 50 years of age [1]. It is defined as the descent of one or more of the following: anterior vaginal wall, posterior vaginal wall, the uterus (cervix), or the apex of the vagina (vaginal vault or cuff scar after hysterectomy), and correlates with symptoms [2].

Current treatment for prolapse consists of surgery, conservative management or “watchful waiting”. Mechanical interventions (such as pessaries) and lifestyle interventions (such as weight loss and avoiding placing strain on the pelvic floor) are both conservative management options. In addition, many women’s health physiotherapists offer pelvic floor muscle training (PFMT). PFMT aims to improve structural support for pelvic organs through effective exercise of the pelvic floor muscles [3], and there is robust evidence of its effectiveness as a treatment for prolapse [4].

Research into the prevention, assessment and effective treatment of prolapse is crucial, as the prevalence is predicted to increase substantially as the population ages. Some predict that the rate of women seeking treatment for prolapse will double over the next 30 years [5]. In 2002, a survey was carried out that reported UK physiotherapy practices in relation to women with prolapse. The survey sought to establish the current use of PFMT in the treatment of prolapse and to provide background information for a planned randomised controlled trial evaluating the effectiveness of PFMT [6]. Questionnaires were mailed to members of the Association of Chartered Physiotherapists in Women’s Health (ACPWH, now renamed “Pelvic, Obstetric and Gynaecological Physiotherapy”, POGP) to investigate the number of women with prolapse treated, treatments offered, guidelines and measures of treatment progress used. The results indicated that PFMT was widely practiced by women’s health physiotherapists throughout the UK, with over 96 % of respondents using the main elements of PFMT during the treatment of prolapse, despite scarce evidence of effectiveness and a lack of guidelines at that time.

Since the publication of this survey, further research has been conducted assessing the effectiveness of PFMT. The Pelvic Organ Prolapse PhysiotherapY (POPPY) multicentre trial [4] reported a greater reduction in prolapse symptoms after 1 year in women who had received PFMT than in those who had not. This trial, two other full-size trials [7, 8], and a number of smaller pilot studies, have begun to establish an evidence base supporting the use of PFMT in the treatment of prolapse [9].

In 2013, a survey was conducted to explore the hypothesis that physiotherapy practice with regard to prolapse would have changed in the last decade in response to the growing evidence base.

The aims were to:

1. Investigate current physiotherapy practice in the treatment of prolapse, specifically PFMT, across the UK;
2. Explore the impact of recent research evidence relating to the conservative management of women with prolapse.

## Materials and methods

The original survey tool, developed in 2002, was updated to gather the necessary data on current physiotherapy practices in the treatment of prolapse across the UK, including changes experienced. In line with Cochrane guidance on the use of electronic surveys [10], the tool was kept short, taking less than 10 min to complete. The survey was accessed via a link sent in an email to the potential participants.

Once the survey link was activated, there was introductory information about the research, followed by a question that asked for the individual’s consent to take part. Of the remaining 19 questions, 5 explored the grade, additional qualifications, experience and place of work of the respondents; 7 questions considered current practice in the assessment and treatment of prolapse; and 7 questions investigated awareness of the POPPY trial and its results. Where appropriate, respondents were able to give free text responses, e.g. “other—please specify”, “please describe”, and “what is the reason?”

In August 2013, the finalised electronic questionnaire was sent to a contact in both ACPWH and the Chartered Physiotherapists Promoting Continence (CPPC), who then distributed the email to the 519 out of 540 members (96 %) who had agreed to receive mailings regarding such surveys.

Each questionnaire was completed online anonymously and participants were asked to respond within 2 weeks of receiving the email. After 2 weeks, a reminder email was distributed to all individuals on the initial mailing list as it was not possible to identify those who had already completed the online survey from those who had not. A final reminder email was sent 2 weeks later, before the survey closed on 25 September 2013.

Data were entered and analysed using the Statistical Package for the Social Sciences (SPSS, version 19). Summary statistics and Chi-squared tests were used to describe data and investigate the association between responses. Ethical approval to undertake the research was granted by Glasgow Caledonian University Departmental Ethics Committee (HLS12/116, 06/08/13).

Respondents were physiotherapists registered with the ACPWH or the CPPC in the UK.

## Results

Of the 519 surveys initially sent, 289 responses were received (56 %). Just over half of responses were received within the first 2 weeks (51 %, *n* = 147) and the two reminder emails prompted an additional 142 responses (49 %). Of the 289 responses, 36 were discarded as all questions were submitted unanswered, which produced a response rate for analysable data of 253 out of 519 (49 %).

The majority of respondents reported that they were senior physiotherapists (55 %, *n* = 128), followed by junior (27 %, *n* = 64) and clinical specialists (18 %, *n *= 42). Fifty-five respondents chose not to indicate their grade (19 %). This result is similar to that of the earlier survey, which reported that the majority of respondents (59 %, *n* = 211) were senior physiotherapists.

Fifty-three percent of respondents (*n* = 132) had been working in women’s health physiotherapy for more than 10 years. A similar proportion indicated 1–5 years (21 %, *n* = 52) and 6–10 years (23 %, *n *= 57) of experience. Very few individuals had worked in women’s health physiotherapy for less than 1 year (3 %, *n* = 8). In the previous survey, there was a broader spread of clinical experience amongst respondents, with 33 % (*n* = 122) working in the field for 5 years or less, 30 % (*n* = 108) had been working between 6 and 10 years and 37 % (*n* = 134) had more than 10 years’ experience.

Over half of respondents had completed a validated continence course (51 %, 128 out of 253), whilst “other” courses completed by almost a third (31 %, 79 out of 253) included advanced bladder and bowel workshops and pelvic floor assessment courses.

Most respondents reported working in the specialty of gynaecology or urogynaecology (Table 1). More than half reported that they worked across three or four different specialities. “Other” specialist areas reported included colorectal, continence, musculoskeletal and sexual health clinics. This result is similar to that of the previous survey in that the majority also reported working in gynaecology.

**Table 1:** Specialities, health settings and prolapse workload of respondents of the previous and the current survey

	2002 survey^a^ frequency (%)	2013 survey frequency (%)
Which speciality do you work within?		
Gynaecology	279 (76.6)	197 (77.9)
Obstetrics	273 (75.0)	171 (67.6)
Urology	173 (47.5)	126 (49.8)
Urogynaecology	230 (63.2)	192 (75.9)
Other	53 (14.6)	98 (38.7)
Which of the following health settings do you work in?		
Primary care	92 (25.3)	59 (23.3)
Outpatients	318 (87.4)	196 (77.5)
Inpatients	208 (57.1)	92 (36.4)
Private practice	81 (22.3)	84 (33.2)
Other—please describe:	11 (3.0)	20 (7.9)
Number of women with prolapse treated in past year		
None	31 (8.5)	8 (3.2)
1–25	164 (45.1)	65 (26.3)
26–50	115 (31.6)	86 (34.8)
51+	52 (14.3)	88 (35.6)

^a^Denominator for 2002 survey is *n* = 364

The majority of respondents reported working in an outpatient care setting and over half reported working across two or more different health settings (Table 1). The “other” health settings reported included community health care and a private hospital. In the previous survey a similar result was reported, with the majority working in outpatient care.

Only 3 % of respondents reported they had not assessed or treated any women with prolapse in the last year, compared with 9 % reported in the previous survey (Table 1). The majority had assessed or treated 26 women or more, and this was significantly more than in the previous survey (Χ^2^ = 49.9, df = 3, *p* < 0.001).

There was a significant association (Χ^2^ = 13.2, df = 6, *p* = 0.04) between the grade and the number of women assessed or treated for prolapse (Table 2). This reflects the results of the 2002 survey, where it was reported that physiotherapists at a higher grade had a higher prolapse caseload.

**Table 2:** Number of women with prolapse assessed or treated in the previous year, by grade of physiotherapist, 2013 survey

Grade	Estimate approximately how many women have you assessed or treated for prolapse within the last year frequency (%)				Total
	None	1–25	26–50	51+	
Junior	5 (8.1)	19 (30.6)	19 (30.6)	19 (30.6)	62 (100.0)
Senior	2 (1.6)	30 (23.6)	51 (40.2)	44 (34.6)	127 (100.0)
Clinical specialist	1 (2.4)	8 (19.5)	10 (24.4)	22 (53.7)	41 (100.0)
Total	8 (3.5)	57 (24.8)	80 (34.8)	85 (37.0)	230 (100.0)

A large majority of respondents reported that the number of women referred to them with prolapse had increased in the last 5 years (70 %, *n* = 157) with a further 26 % (*n *= 59) reporting “no change”. Only 4 % (*n* = 10) reported that the number of women referred to them had decreased over the last 5 years. When asked what the reason was for any change, respondents speculated that it might be in response to an increased awareness of prolapse and also an increased awareness among GPs concerning the benefits of physiotherapy in the treatment of the condition.

Of those respondents who had assessed or treated women with prolapse, the majority took referrals from gynaecology and primary health care (Table 3). In the previous survey too, the majority reported taking referrals from gynaecology; however, this percentage was even greater. In the current survey, two thirds of respondents reported receiving referrals from between three and five different specialities. “Other” referral sources included colorectal clinics, health visitors and midwives.

**Table 3:** Source of referral of women for assessment and treatment, and treatments offered for pelvic organ prolapse

	2002 survey frequency (%)	2013 survey^a^ frequency (%)
Where were women with prolapse referred from?		
Primary care (GP)	248 (74.5)	189 (74.7)
Community continence service		69 (27.3)
Urology	136 (40.8)	62 (24.5)
Urogynaecology		163 (64.4)
Gynaecology	310 (93.1)	195 (77.1)
Obstetrics	187 (56.3)	116 (45.8)
Self-referred		107 (42.3)
Other	38 (11.4)	32 (12.6)
What treatment might you offer women with prolapse?		
PFMT (one-to-one)	322 (96.7)	234 (92.5)
PFMT (classes)		38 (15.0)
Lifestyle advice (e.g. lifting, losing weight)		232 (91.7)
Vaginal cones	117 (35.1)	38 (15.0)
Biofeedback-assisted PFMT	277 (83.2)	209 (82.6)
Biofeedback—verbal		178 (70.4)
Biofeedback—EMG		147 (58.1)
Biofeedback—educator		119 (47.0)
Biofeedback—manometry		16 (6.3)
Electrical stimulation	252 (75.7)	169 (66.8)
Pessary		28 (11.1)
Pessary + PFMT		49 (19.4)
Other	52 (15.9)	52 (20.6)

*PFMT* pelvic floor muscle training, *EMG* electromyography

^a^Denominator for 2002 survey is *n* = 333

Respondents offered a range of treatments to women with prolapse. The most commonly offered treatments were one-to-one PFMT and lifestyle advice (Table 3). Biofeedback-assisted PFMT was another treatment offered by the majority in both the previous survey and the current survey. The use of vaginal cones as a treatment was less commonly reported in the current survey. Treatments such as the use of a pessary, pessary + PFMT and group-delivered PFMT were also reported by respondents to a lesser extent in this survey. These treatment options were not specifically investigated in the earlier survey. “Other” treatments offered included “referral to a specialist to fit pessary” and “bowel and bladder advice”. The most common number of PFMT appointments usually offered was four (Table 4).

**Table 4:** Average number of PFMT appointments offered by respondents to women with prolapse^a^

Average number of PFMT appointments	Frequency	Percentage
2	11	3.8
3	49	17.0
4	80	27.7
5	41	14.2
6	27	9.3
7	5	1.7
8	2	0.7
9	1	0.3

^a^Exact figures reported where possible. When a range was given, the mean was used, and rounded up to a whole number (e.g. for “2–5 appointments” the mean of 3.5 was rounded to 4)

Almost half of respondents reported that they did not have access to guidelines outlining what treatments should be offered for prolapse. More than half did not have access to guidelines stating which women should be offered prolapse treatment and most did not have access to a prolapse care pathway. Those who reported having access to guidelines cited the National Institute for Health and Care Excellence (NICE), Chartered Society of Physiotherapy (CSP), Royal College of Obstetricians and Gynaecologists (RCOG) or local guidelines.

In line with the earlier survey, pelvic floor muscle (PFM) strength was the most common measure used to monitor patient progress (Table 5). Methods for grading the severity of prolapse were used by a smaller group of respondents compared with the 2002 survey. Measures such as the Pelvic Floor Distress Inventory (PFDI), the Pelvic Floor Impact Questionnaire (PFIQ) and the Electronic Personal Assessment Questionnaire (EPAQ) were also used by a few respondents. The International Consultation on Incontinence Questionnaire (ICIQ) Vaginal Symptoms Score and the Pelvic Organ Prolapse Symptom Score (POP-SS) were the two most commonly reported symptom measures used. “Other” measures used included the Urogenital Distress Inventory and re-assessing patient symptoms.

**Table 5:** Measures used by respondents to monitor the progress of women with prolapse

Do you use any of the following measures to monitor patient progress?	2002	2013^a^
	Frequency (%)	Frequency (%)
Prolapse grading	148 (44.4)	
POP-Q		44 (17.8)
Other staging measure (e.g. Baden & Walker)		11 (4.3)
PFM strength (e.g. Oxford scale, ICS method)	313 (94.0)	202 (80.2)
Patient reported symptoms	324 (97.3)	
ICIQ Vaginal Symptoms Score		82 (32.8)
MYMOP		5 (2.0)
POP-SS		59 (23.3)
PFDI		12 (4.7)
EPAQ		7 (2.8)
Quality of life	280 (84.1)	5 (2.0)
P-QoL		23 (9.1)
PFIQ		8 (3.2)
Other	23 (6.3)	42 (16.6)

*POP-Q* Pelvic Organ Prolapse Quantification, *PFM* pelvic floor muscles, *ICIQ* International Consultation on Incontinence Questionnaire, *MYMOP* Measure Yourself Medical Outcome Profile, *POP-SS* Pelvic Organ Prolapse Symptom Score, *PFDI* Pelvic Floor Distress Inventory, *EPAQ* Electronic Personal Assessment Questionnaire, *P-QoL* Perceived Quality of Life, *PFIQ* Pelvic Floor Impact Questionnaire

^a^Denominator for 2002 survey is *n* = 333

A large majority of respondents were aware of the POPPY randomised controlled trial (80 %, *n *= 190) and 11 % (*n* = 20) were involved in the trial. Sixty-eight percent of respondents (*n* = 128) were aware of the published results, which indicated that PFMT was effective for reducing prolapse symptoms, and almost half (48 %, *n *= 59) reported that these results had made them change their practice. Specific reasons behind the change in practice were an increased knowledge of the capabilities of PFMT or increased confidence in suggesting this as an effective treatment. The majority (56 %, *n* = 67), however, believed that there were barriers to implementing the delivery of PFMT for prolapse, including: scepticism amongst medical practitioners concerning the benefits of physiotherapy; GPs referring patients for surgery as a first-line treatment before considering conservative therapy options; and “limited resources”.

## Discussion

This survey investigated current physiotherapy practice in the treatment of prolapse and the impact that recent research has had on the physiotherapeutic management of women with the condition. The responses supported the hypothesis that practice had adapted in line with the evidence base. An increased number of women were being referred to physiotherapists for assessment and treatment of prolapse, from a greater range of sources, compared with 10 years ago. One-to-one PFMT was reported to be the most common conservative treatment for the condition, with lifestyle advice and biofeedback-assisted PFMT also very frequently used. The majority of respondents were aware of evidence from a definitive trial to support the effectiveness of PFMT in the treatment of prolapse, and almost half had used the results to alter their practice.

To our knowledge there are no other surveys with which to compare our findings. Two other surveys of UK prolapse practice have been published, but have focused only on surgical treatment [11, 12].

The respondents of this survey did not only report seeing a greater number of women with prolapse than in the initial survey, but also an increase in the number over the last 5 years. When asked to identify reasons for this increase in numbers, respondents overwhelmingly cited “increased awareness” as the cause. It was reported that awareness within the general public about the condition had increased as had practitioners’ awareness of the role of physiotherapy as a treatment. It was also highlighted that increased evidence and the changing opinions of practitioners were resulting in a greater number of referrals to physiotherapists. The respondents also reported that changing care pathways had led to an increased number of women being referred to physiotherapy before being considered for surgery. A few respondents mentioned the negative impact that the recent media coverage regarding surgery and mesh implants has had. The responses from the current survey support those from the 2002 survey in that the physiotherapists of a higher grade tended to report assessing or treating a higher number of patients. This may be because the prolapse patient might present with co-existing vaginal, urinary, bowel and sexual symptoms, and thus treatment requires a greater breadth of knowledge and clinical experience.

Recent research provides evidence of the beneficial effects of PFMT on prolapse, and almost half of respondents had changed their practice as a result. The introduction into practice of a wider variety of treatments such as group PFMT and pessaries was also evident, as was the combination of PFMT with a pessary.

Pelvic floor muscle strength was still the most commonly used measure of treatment progress. It was encouraging that 18 % of respondents were using the POP-Q [13], as the reliability of physiotherapists using the POP-Q has recently been demonstrated to be good [14], and the availability of such data is useful for multidisciplinary discussion. A survey of gynaecologists and urogynaecologists, who may be more established in their use of the POP-Q, found that 40 % used the POP-Q in routine clinical practice [15].

There was considerable use of validated symptom measures, in particular the POP-SS [16] and the ICIQ Vaginal Symptoms Score [17]. Such symptom outcome measures are valuable as symptoms are the main driver for women seeking treatment.

Respondents using guidelines named the National Institute for Health and Care Excellence (NICE), Royal College of Obstetricians and Gynaecologists (RCOG) and Chartered Society of Physiotherapy (CSP) guidelines. NICE propose care pathways for urinary incontinence in women, advise pelvic floor exercises in the treatment of stress urinary incontinence, and offer guidelines on surgical mesh repair in the treatment of prolapse. However, there are no care pathways, clinical guidelines or referral guidelines specific to prolapse. RCOG offer only guidelines on “post-hysterectomy vaginal vault prolapse”, which state that the role of conservative measures in treatment, including pelvic floor exercise, is “unclear”. The CSP offers no guidelines of its own, but recommends using those produced by NICE. Although not mentioned by respondents, the International Consultation on Incontinence textbook, which synthesises the evidence on incontinence and other pelvic floor problems, provides a treatment pathway for prolapse that includes a recommendation for PFMT before surgical intervention [18]. The need for clear, accessible guidelines and care pathways is apparent and will become increasingly important in avoiding large variations in local practice primarily determined by the practitioners’ individual experience.

As awareness of the evidence supporting the use of PFMT continues to grow, it is likely that more practitioners will refer women for physiotherapy and more physiotherapists will be required to deliver PFMT. Over half of respondents had spent more than 10 years working in the field of women’s health. The future inevitable loss of this vast experience and knowledge of these senior practitioners needs to be planned for. Pathways into women’s health for physiotherapists in the UK are limited and need to be considered.

Respondents were working in a wide range of different specialties and receiving referrals from a range of sources, highlighting how widespread this problem is. This further stresses the need for resources and awareness to manage the expected increase in the prevalence of prolapse within the population [5].

Although the results of this survey are similar to those found in the earlier survey carried out by the authors, the response rate here was lower. This may be accounted for by the different distribution method used in the recent survey: email as opposed to postal delivery. Whilst email is a faster method of communication, an email may have been easier to overlook or seemed less personal, making respondents less inclined to respond. Contrary to this, studies comparing email and postal administration of surveys to healthcare professionals have indicated that emails are preferred by recipients, leading to higher response rates [19, 20]. Our response rate of 49 % is, however, similar to the mean response rate of 53 % found from across 490 published surveys [21].

Despite the lower response rate, the demographics of the respondents from 2002 and 2013 were similar in that the majority were senior physiotherapists, and the majority reported working in gynaecology and saw women in outpatient care. Thus the profiles of the two samples are similar.

The sampling frame for the survey was the mailing lists of the ACPWH and CPPC professional organisations. Although there are women’s health physiotherapists who are not members of these professional organisations, and therefore would not have received this survey directly, we asked respondents to pass on the survey link to colleagues who were non-members. In addition, the survey link was distributed at the ACPWH annual conference where 18 % of attendees were non-members.

In conclusion, the survey results indicate that there is awareness, and has been uptake, of evidence-based practice in the last decade, and PFMT is being delivered to greater numbers of women with prolapse by the majority of specialist physiotherapists. Barriers to further implementation, such as a lack of trained staff to deliver PFMT and the lack of guidelines, need to be addressed to support the growing need for conservative treatment.
